# Sex Differences in the Hepatic Cholesterol Sensing Mechanisms in Mice

**DOI:** 10.3390/molecules180911067

**Published:** 2013-09-10

**Authors:** Gregor Lorbek, Martina Perše, Simon Horvat, Ingemar Björkhem, Damjana Rozman

**Affiliations:** 1 Center for Functional Genomics and Bio-Chips, Institute of Biochemistry, Faculty of Medicine, University of Ljubljana, SI-1000 Ljubljana, Slovenia; E-Mail: gregor.lorbek@mf.uni-lj.si; 2 Medical Experimental Centre, Institute of Pathology, Faculty of Medicine, University of Ljubljana, SI-1000 Ljubljana, Slovenia; E-Mail: martina.perse@mf.uni-lj.si; 3 Department of Animal Science, Biotechnical Faculty, University of Ljubljana, SI-1000 Ljubljana, Slovenia; E-Mail: simon.horvat@bf.uni-lj.si or simon.horvat@ki.si; 4 National Institute of Chemistry, SI-1000 Ljubljana, Slovenia; 5 Department of Laboratory Medicine, Division of Clinical Chemistry, Karolinska Institute, Karolinska University Hospital, SE-141 86 Huddinge, Sweden; E-Mail: ingemar.bjorkhem@karolinska.se

**Keywords:** cholesterol synthesis, high-fat diet, gene expression, sterol, bile acid, liver, mouse

## Abstract

Cholesterol is linked to many multifactorial disorders, including different forms of liver disease where development and severity depend on the sex. We performed a detailed analysis of cholesterol and bile acid synthesis pathways at the level of genes and metabolites combined with the expression studies of hepatic cholesterol uptake and transport in female and male mice fed with a high-fat diet with or without cholesterol. Lack of dietary cholesterol led to a stronger response of the sterol sensing mechanism in females, resulting in higher expression of cholesterogenic genes compared to males. With cholesterol in the diet, the genes were down-regulated in both sexes; however, males maintained a more efficient hepatic metabolic flux through the pathway. Females had higher content of hepatic cholesterol but this was likely not due to diminished excretion but rather due to increased synthesis and absorption. Dietary cholesterol and sex were not important for gallbladder bile acids composition. Neither sex up-regulated *Cyp7a1* upon cholesterol loading and there was no compensatory up-regulation of *Abcg5* or *Abcg8* transporters. On the other hand, females had higher expression of the *Ldlr* and *Cd36* genes. These findings explain sexual dimorphism of cholesterol metabolism in response to dietary cholesterol in a high-fat diet in mice, which contributes to understanding the sex-basis of cholesterol-associated liver diseases.

## 1. Introduction

Sexual differentiation is one of the principal features of our species and is driven by the sex hormones, namely androgens in males and estrogens in females. In spite of this, gender differences were frequently neglected in the past, when most epidemiological and also mechanistic studies were performed only in males and the conclusions then simply extrapolated to females irrespective of the fundamental biological disparities between them [1]. Nevertheless, there is increasing evidence of sex- bias in several fields, such as cardiovascular diseases (CVD) [2], diabetes [3], autoimmune diseases [4], dyslipidemia [5] and liver diseases [6], to name just a few. For example, women are more protected against hypercholesterolemia until menopause and thus have lower incidence of CVD than men. Managing the imbalances of cholesterol homeostasis with hypolipidemic drugs represents the corner stone of today’s CVD therapy [7], with differing efficacy in relation to the gender [8]. Recently, aberrant cholesterol synthesis has also been implicated as one of the putative driving factors of non-alcoholic fatty liver disease (NAFLD) [9], which has been recognized as an important and self-governing risk for developing CVD [10]. Prevalence of NAFLD is sexually dimorphic with the majority of epidemiological data showing a clear male predominance [11,12,13], yet few studies suggest that women are at higher risk of developing NAFLD than men [14,15]. Part of the bias might arise from a variety in the number of participants, their age, ethnic group and in measures of diagnosing the disease. Scarce studies on animal models are also not conclusive, some indicating male- [16] and other female-bias [17,18]. Comhair *et al.* [18] showed that dietary and plasma cholesterol correlate with liver inflammation, especially in the female mice. These results, however, are not easily applied to humans, since animal models do not fully reflect the metabolic context, pathology and etiology of NAFLD [19]. Mice have fundamentally different cholesterol metabolism than primates [20], which depends also upon the genetic background of the animal [21].

Many rodent studies that focused on the rate-limiting enzyme 3-hydroxy-3-methylglutaryl-Coenzyme A reductase (HMGCR), the LDL receptor (LDLR), or sterol regulatory element binding protein, type 2 (SREBP-2) as the major cholesterol-related transcriptional regulator [5,22,23,24,25], did identify sex differences in disruption of cholesterol homeostasis and disease outcome (e.g., in NAFLD). More recent studies investigated liver sex differences at the level of whole transcriptome and identified lipid metabolic pathways (fatty acid, cholesterol and triglyceride metabolism) as being sex-biased [26,27,28,29,30]. Also here major discrepancies exist between the studies. Apart from the differences in the genetic background and the experimental design (age, length of treatments, *etc.*), composition of the applied diets represents another variable. Cholesterol was present to a different degree in many formulations, because it is found, for example, in lard and casein.

Our broader interest is to understand the mechanisms of sex-related differences in healthy and diseased liver. In this study we investigated sex differences in hepatic cholesterol biosynthesis and metabolism in mice of mixed genetic background (129/Pas × C57BL/6J, close to 90% of C57BL/6J) using purified diet at both extremes of cholesterol content (either no cholesterol or 1.25% cholesterol) in a high-fat background. As rodents do not have a specific requirement for cholesterol in the diet, this allowed us to precisely evaluate sex differences in murine hepatic cholesterol sensing mechanisms.

## 2. Results and Discussion

### 2.1. Expression of Cholesterogenic Genes in Livers of Male and Female Mice

**Figure 1:**
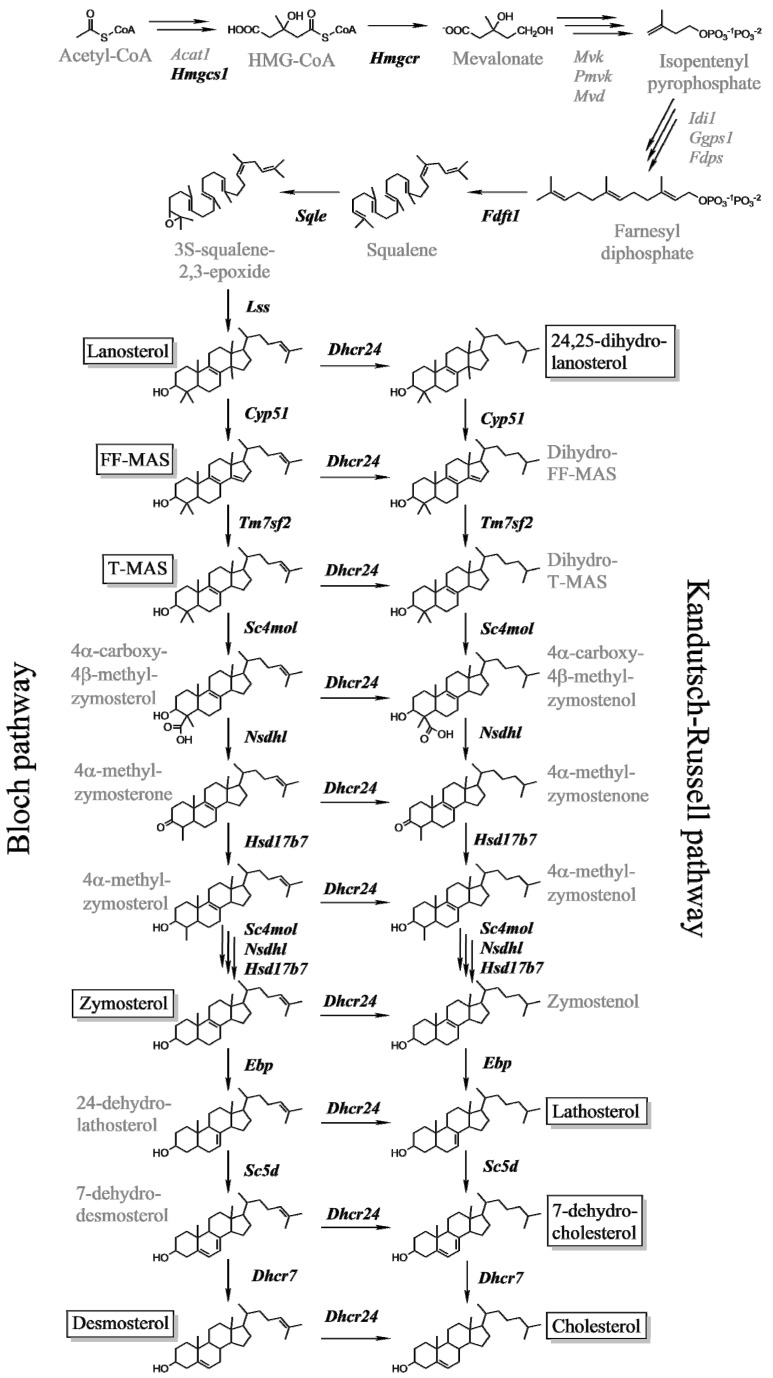
Cholesterol synthesis scheme. The investigated genes are in black bold text and the measured intermediates are in framed black text. Adapted after [31].

We measured the expression of 14 genes from cholesterol synthesis in the livers of females and males on a high-fat diet with (HFC) or without (HFnC) cholesterol. All investigated genes are depicted on Figure 1. Initially we investigated males and females together to evaluate the sex-pooled hepatic effects. Addition of 1.25% of cholesterol to the high-fat diet significantly down-regulated mRNA expression of the entire cholesterol synthesis (Supplementary Table S2). Genes with the highest suppression were *Sc4mol* (Fold change (FC) = 0.06), *Sqle* (FC = 0.07), *Nsdhl* (FC = 0.10) and *Hmgcs1* (FC = 0.10), followed by *Hmgcr* (FC = 0.16) and *Cyp51* (FC = 0.18). The least down-regulated was *Dhcr24* (FC = 0.47), encoding 24-dehydrocholesterol reductase, which splits the biosynthesis pathway into the Bloch and Kandutsch-Russell branches.

**Figure 2:**
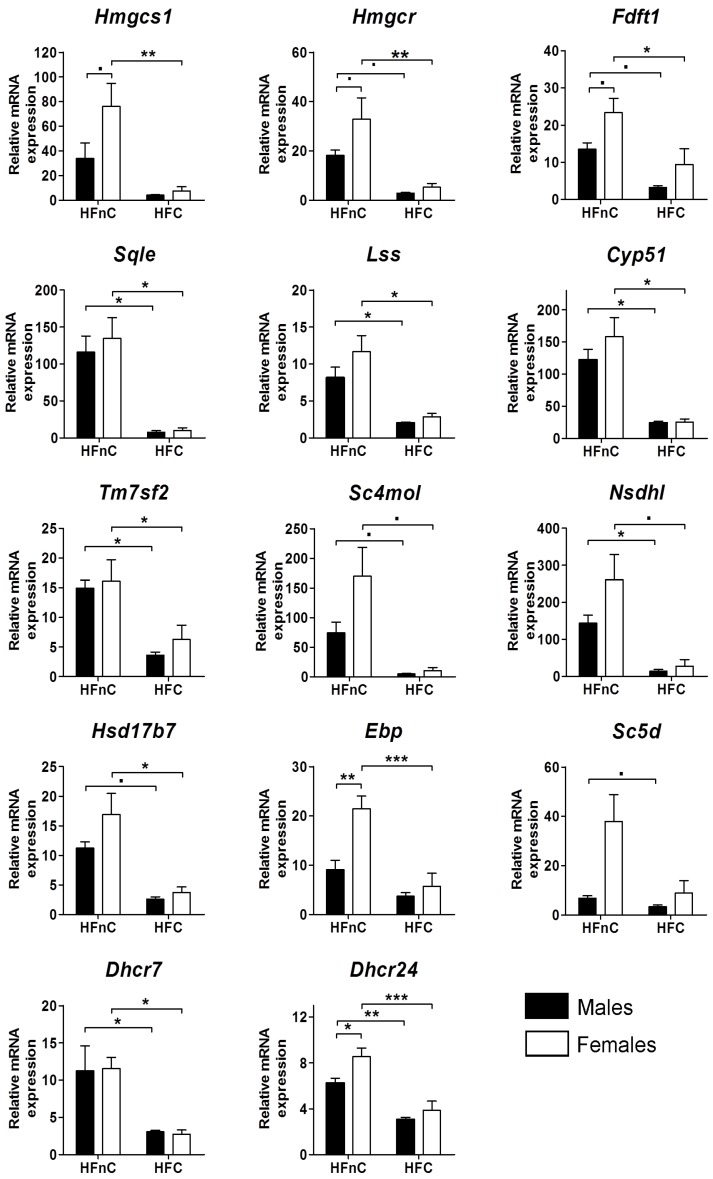
Relative mRNA expression of cholesterogenic genes in the liver. Black columns represent male and white columns female mice. Error bars represent SEMs. N = 5; **˙** *p* < 0.1; * *p* < 0.05; ** *p* < 0.01; *** *p* < 0.001.

If we analyzed each sex separately, *Fdft1* (FC_M vs F_ = 0.24 *vs.* 0.40) and *Tm7sf2* (FC_ M vs F_ = 0.24 *vs.* 0.39) got more suppressed in males while *Ebp* (FC_M vs F_ = 0.41 *vs*. 0.27) and *Sc5d* (FC_M vs F_ = 0.49 *vs.* 0.23) more in females, whereas other genes were down-regulated to roughly the same extent in both sexes (Figure 2 and Supplementary Table S2).

Taking a closer look at the differences between the sexes on individual diets, we can observe a consistent trend of higher expression of cholesterogenic genes in females on HFnC diet (FC > 1), even though the significance for various genes did not reach the statistical threshold. Expression of *Dhcr7*, that encodes 7-dehydrocholesterol reductase for the conversion of 7-dehydrodesmosterol and its analogue 7-dehydrocholesterol to cholesterol, stayed the same in males and females. Statistically most significant differences between the sexes were in the expression of *Dhcr24* (*p* = 0.045) and *Ebp* (*p* = 0.004) on the HFnC diet, nevertheless, females had, on average, higher expression of the majority of cholesterogenic genes.

### 2.2. Hepatic Metabolites of Cholesterol Synthesis

Hepatic contents of cholesterol and its precursors were determined by gas chromatography-mass spectrometry (GC-MS) that enabled quantification of several structurally similar intermediates from cholesterol synthesis pathway in a single run (Figure 1). Cholesterol was analyzed separately because liver contains about three orders of magnitude more total cholesterol than the intermediates.

**Figure 3:**
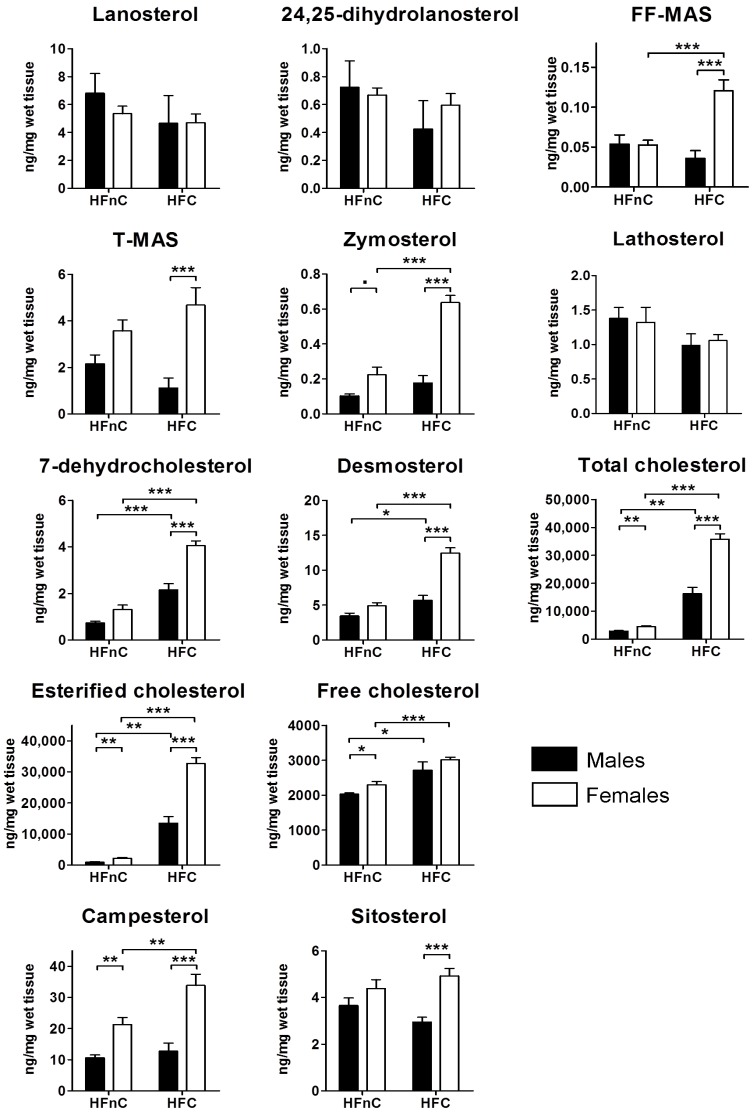
Hepatic contents of cholesterol, cholesterol intermediates and plant sterols expressed as ng/mg of wet liver tissue. Black columns represent male and white columns female mice. Error bars represent SEMs. N = 9–10; **˙** *p* < 0.1; * *p* < 0.05; ** *p* < 0.01; *** *p* < 0.001.

Levels of lanosterol and 24,25-dihydrolanosterol, the first two sterol intermediates, were unaffected by dietary cholesterol or sex. In the post-lanosterol part of the pathway, the most prominent observation was that females on HFC diet had significantly higher amounts of all investigated intermediates compared to the males, with the exception of lathosterol (Figure 3).

Females also showed higher amounts of cholesterol intermediates when cholesterol was added to the high-fat diet (HFnC compared to HFC); however, no change was noted in the amounts of T-MAS and lathosterol. Interestingly, in males dietary cholesterol exerted no effect up to the very last step in cholesterol synthesis: quantities of 7-dehydrocholesterol and desmosterol were increased in the HFC diet treatment (Figure 3).

As expected, addition of cholesterol to the diet resulted in a vast increase of hepatic cholesterol content, mostly in its esterified form. Females had higher amounts of hepatic total and esterified cholesterol than males on both diets. Similar differences were observed also for free cholesterol, although statistical significance between the sexes was achieved on HFnC diet treatment only (Figure 3).

Campesterol and sitosterol, two plant sterols that are absorbed together with cholesterol and are most abundantly accumulating in the liver were also quantified. Dietary cholesterol did not influence hepatic sitosterol and campesterol amounts in males. Female mice on HFC diet had increased levels of campesterol but not sitosterol when compared to the HFnC diet. The sex effect was highly significant and plant sterols were higher in females on both types of diet with the exception of sitosterol on the HFnC diet (Figure 3).

### 2.3. Expression of Hepatic Genes of Cholesterol Regulation and Transport

**Figure 4:**
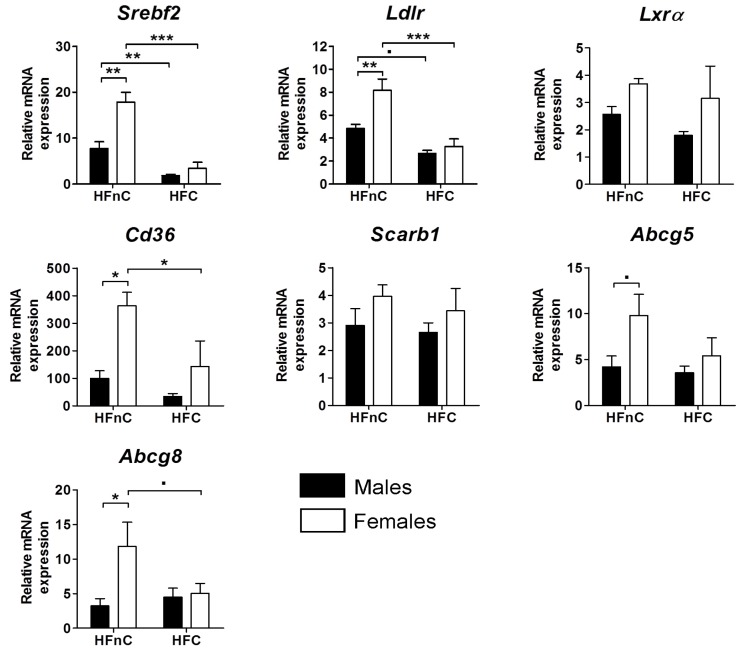
Relative mRNA expression of cholesterol-related genes in the liver. Black columns represent male and white columns female mice. Error bars represent SEMs. N = 5; **˙** *p* < 0.1; * *p* < 0.05; ** *p* < 0.01; *** *p* < 0.001.

In addition to investigating cholesterol synthesis pathway, we ascertained the expression of certain key genes that are closely linked to cholesterol metabolism in the liver (Figure 4). Cholesterol synthesis is tightly regulated *via* a sterol sensing feedback mechanism [32] that activates the transcription of target genes by SREBP-2.

Our results show a suppression of *Srebf2* by dietary cholesterol in both sexes; however, females on HFnC diet had significantly higher expression of this gene than males. LDL receptor (*Ldlr*) is also a target of SREBP-2 and is regulated by the same negative feedback loop. When intracellular levels of cholesterol are depleted, the expression of *Ldlr* is induced in order to increase the LDL cholesterol clearance from the plasma [33]. The expression pattern of *Ldlr* in our mice closely resembled that of *Srebf2*: a female-biased expression on HFnC diet and a suppression effect of dietary cholesterol in both sexes was observed. The next investigated gene was *Lxrα* encoding the liver X receptor (LXR) that is involved in cholesterol homeostasis [34]. Our results show that *Lxrα* was not subjected to regulation by either dietary cholesterol or sex. ABCG5 and ABCG8 are members of the ATP transporter family that form heterodimers and promote biliary excretion of cholesterol and plant sterols. Females on HFnC diet had higher expression of both transporters than males. It was reported that high cholesterol synthesis or dietary intake promotes transcription of these two genes *via* LXRs in order to efficiently remove excess cholesterol from the liver [35]. Surprisingly, our data show that dietary cholesterol in males did not influence the expression of *Abcg5* and *Abcg8*, whereas in females the addition of cholesterol even reduced the expression of both transporters.

We also measured the expression of the scavenger receptor class B type 1 (SR-BI) that is responsible for selective uptake of HDL cholesterol to the liver and has recently been described as a mediator of biliary cholesterol secretion [36]. *Scarb1* expression was not affected by dietary cholesterol neither it was sex-biased. Another investigated type of scavenger receptor was fatty acid translocase/CD36 (FAT/CD36) with broad substrate specificity and an active role in reverse cholesterol transport and long-chain fatty acid uptake [37]. Expression of *Cd36* was higher in females compared to males, whereas addition of dietary cholesterol reduced the expression only in females.

### 2.4. Bile Acid Synthesis Genes and Metabolites

**Figure 5:**
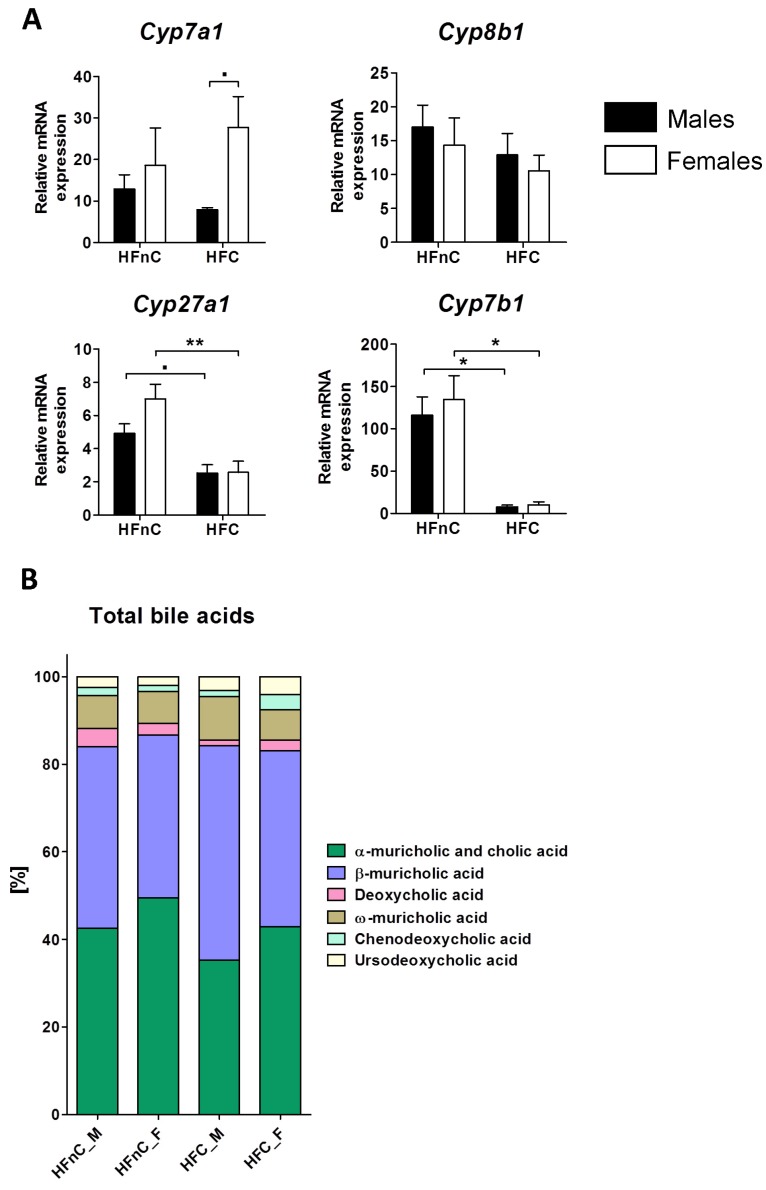
Expression of bile acid synthesis genes (A) in the liver and gallbladder bile acid composition (B). Black columns represent male and white columns female mice. Error bars represent SEMs. N = 8; **˙** *p* < 0.1; * *p* < 0.05; ** *p* < 0.01; *** *p* < 0.001.

To get a better understanding of potential sex-biased processes downstream of cholesterol, we investigated the relative composition of gallbladder bile acids (BA) together with key cytochrome P450 genes from their synthesis. Interestingly, dietary cholesterol had an impact mainly on the expression of *Cyp27a1* and *Cyp7b1* from the alternative BA synthesis pathway where both sexes experienced down-regulation to the same extent. Expression of *Cyp7a1*, the rate-limiting step in BA synthesis, tended to be higher in females than males on HFC diet (*p* = 0.09) and females showed a trend of increased *Cyp7a1* expression in the cholesterol loaded diet (Figure 5A).

Dietary cholesterol or sex did not have a major impact on the composition of gallbladder bile acids (Figure 5B and Supplementary Figure S1). Female mice had a higher percentage of chenodeoxycholic and ursodeoxycholic acid on HFC compared to HFnC diet and males had a lower percentage of deoxycholic acid when compared to the HFnC diet. The most prominent difference between males and females was chenodeoxycholic acid on HFC diet, which was higher in females (Supplementary Figure S1).

### 2.5. Discussion

In order to understand the underlying physiological sex differences as possible starting points for progression of cholesterol-associated diseases, we investigated hepatic cholesterol synthesis of 129/Pas × C57BL/6J mice fed a high-fat diet with no cholesterol or with cholesterol supplementation. Mice of both sexes were on the diet for 16 weeks which represents a more relevant model in terms of human diet as compared to the short term feeding (1 to 3 weeks) used in most rodent studies [38]. However, differences in lipid metabolism between rodents and humans need to be taken into account.

#### 2.5.1. Female-Biased Expression of Cholesterogenic Genes is Diminished upon Addition of Dietary Cholesterol

Female mice on HFnC diet had a consistently higher expression of cholesterogenic genes than their male counterparts. Expression of *Dhcr24* and *Ebp* was most significantly different (Figure 2). The majority of other genes were also female-biased, albeit the statistical significance was not achieved. The reason could be that the female mice were not synchronized in their estrous cycles (observed at euthanasia by assessing gross edematous distension of uterine horns according to [39]) and estrogen is supposedly an important regulator of cholesterol metabolism [25]. Statistical power might also be increased by including more samples in qPCR studies. Female mice on HFnC diet had significantly higher expression of *Srebf2* and SREBP2 up-regulates all cholesterogenic genes [40]. Lack of dietary cholesterol in the high-fat diet seems to result in a stronger response of the sterol sensing feedback loop in females resulting in higher expression of cholesterol synthesis genes. In contrast, when cholesterol was added to the diet, the expression was down-regulated to roughly the same extent in both sexes. In these conditions the daily needs are easily met either from dietary cholesterol [41] or from reverse cholesterol transport.

Transcriptome studies of mouse liver provide further evidence that the expression of cholesterogenic genes is sex-biased. Gatti *et al.* [28] identified higher expression of *Hmgcs1*, *Fdft1*, *Sqle*, *Lss*, *Tm7sf2*, *Ebp*, *Sc5d*, *Dhcr24* and *Srebf2* in females. Another study has shown that *Hmgcr* and *Fdft1* were female-biased, but *Cyp51*, *Sc4mol*, *Hsd17b7*, *Dhcr7* and *Dhcr24* were male-biased [27]. Genome-wide analysis of mouse developmental changes established *Hmgcs1*, *Fdft1* and *Dhcr24* as being female-biased at the age of 8 weeks [26]. The sex-bias of cholesterol synthesis remains inconsistent, which could probably be ascribed to the use of mouse models with different genetic backgrounds, diets or treatment regimes. Nevertheless, in concert with our study, cholesterol synthesis seems to be sex-dependent with the majority of evidence pointing towards higher expression of cholesterogenic genes in genetically non-modified female mice.

#### 2.5.2. Dietary Cholesterol in High-Fat Diet Down-Regulates the Entire Cholesterol Synthesis Pathway

Upon addition of cholesterol to the high-fat diet the entire cholesterol synthesis gets down-regulated. Most suppressed are *Sc4mol*, *Sqle*, *Nsdhl*, *Hmgcs1*, *Hmgcr* and *Cyp51*, which is in agreement with other studies [29,42]. HMGCR is the rate-limiting enzyme of cholesterol synthesis, so strong suppression of its transcription by dietary cholesterol would be expected, but its activity is regulated also post-transcriptionally [43]. SQLE has been proposed as the second rate-limiting enzyme in cholesterol synthesis since addition of cholesterol suppressed *Sqle* and *Hmgcr* mRNAs to the same extent [44]. Given the fact that in our study practically all cholesterogenic genes were suppressed by cholesterol, the cholesterol-dependent transcriptional regulation seems to be in favor of multiple rather than one or two control points, since in this way the most effective metabolic flux through the pathway can be achieved [45]. It has to be taken into account, however, that gene expression does not always reflect protein levels or activity.

#### 2.5.3. Female Mice Have Higher Hepatic Pool of Cholesterol and Post-Lanosterol Synthesis Intermediates

Feeding the mice with cholesterol-enriched diet resulted in increased hepatic total, free and esterified cholesterol in both sexes in agreement with previous findings [29]. Interestingly, females had higher amounts of the three cholesterol species on both diets. Turley *et al.* [46] showed that females have a more efficient absorption of cholesterol from the intestines due to 60% larger, more hydrophobic BA pool. More efficient reabsorption of dietary and biliary cholesterol could explain larger amounts of cholesterol in females on the HFC and partially on the HFnC diet. In line with this are also higher levels of plant sterols that are absorbed from the intestines together with cholesterol [47]. Larger BA pool size alone, however, would probably not result in higher hepatic cholesterol in females on HFnC diet where no dietary cholesterol is present. We hence suggest that elevated cholesterol synthesis also contributed to the expansion of the hepatic cholesterol pool as indicated by female-biased expression of cholesterogenic genes. Furthermore, female mice had higher expression of *Ldlr* and *Cd36* that would suggest increased uptake of cholesterol from the circulation. Our results imply that females have a more active hepatic cholesterol metabolism on HFnC diet than males, which manifests in net accumulation of cholesterol in their livers. In the presence of dietary cholesterol differences were even more prominent, likely due to a more efficient cholesterol absorption from the intestines, since cholesterol synthesis and hepatic uptake pathways were suppressed. Both sexes did also not up-regulate *Cyp7a1* to increase BA synthesis (only a trend in females) and there was no compensatory up-regulation of *Abcg5* or *Abcg8* transporters. *Cyp7a1* regulation in mice seems to depend on the type of dietary fat [48], the duration of feeding and the percentage of cholesterol in the diet [38].

With the exception of lathosterol, the cholesterol biosynthesis intermediates beyond lanosterol and 24,25-dihydrolanosterol were higher in females than males on the HFC diet. Due to a defined chemical composition of the diet, it is unlikely that the cholesterol intermediates would arise from the diet [49]. It thus seems that only the pre-lanosterol part of cholesterol synthesis is regulated equally in both sexes. Males seem to maintain a more efficient metabolic flux through the cholesterol pathway under dietary cholesterol load. It is possible that higher hepatic cholesterol content in females mediates the cholesterol-dependent proteasomal degradation of enzymes, as was shown for SQLE [44,50]. Cholesterol intermediates themselves could also modulate cholesterol synthesis [31]. 7-dehydrocholesterol mediates proteolysis of HMGCR [51] and desmosterol interfered with SREBP-2 processing and reduced the expression of *Hmgcr* [52]. Given the multitude of reactions in cholesterol synthesis and the complexity of regulation, further explanations would warrant more comprehensive approaches such as modeling the metabolic fluxes [53].

#### 2.5.4. Dietary Cholesterol or Sex Do Not Impact Gallbladder Bile Acid Profile

We show that dietary cholesterol and sex are not important for modulating the composition of gallbladder bile acids. The addition of cholesterol to the diet increased the relative amounts of ursodeoxycholic and chenodeoxycholic acid in females and decreased deoxycholic acid in males (Figure 5B). Ursodeoxycholic acid has certain therapeutic effects [54] and together with deoxycholic acid inhibits uptake of long chain fatty acids in hepatocytes [55]. The two secondary bile acids are formed by intestinal bacteria and the physiological significance of intestinal biotransformations is still unknown [56]. The fact that dietary cholesterol down-regulated alternative pathway of BA synthesis could result in altered amounts of chenodeoxycholic acid. However, in mice most of chenodeoxycholic acid is converted to α- and β-muricholic acid, which is the reason for its low abundance in the total bile acid pool.

## 3. Experimental

### 3.1. Animals and Diets

All the procedures on animals were conducted in accordance with the European Convention for the protection of vertebrate animals used for experimental and other scientific purposes (ETS 123) and were approved by Veterinary Administration of the Republic of Slovenia.

Nineteen male and 18 female mice of mixed genetic background (129/Pas × C57BL/6J, close to 90% C57BL/6J) were bred at Medical Experimental Centre, Ljubljana, Slovenia, and maintained in a temperature and humidity controlled environment under 12h light/dark cycle (lights were on from 7.00 am to 7.00 pm). All animals had *ad libitum* access to food and acidified tap water (pH = 3). After 3 weeks on standard laboratory diet (1310, Altromin, Lage, Germany), mice of both sexes were divided in half (n = 9–10) and put on two identical and isocaloric high-fat diets for another 16 weeks. One of the diets contained 1.25% (w/w) of cholesterol (D12108C, Research Diets, Inc., New Brunswick, NJ, USA), whereas the other diet was without cholesterol (D12106C, Research Diets, Inc.).

### 3.2. Sample Collection

Mice were euthanized after a 5 h fast with cervical dislocation at the same time of the light period (12.30 am–2.30 pm) in order to avoid diurnal variability as much as possible. Livers were excised, weighted and bile was removed from gallbladder with a syringe. Livers were then cut to thin slices and snap frozen in liquid N_2_ together with bile. All the material was stored at −80 °C for subsequent analysis.

### 3.3. RNA Isolation and cDNA Synthesis

RNA from the liver was isolated using QuickGene Tissue Kit S II and QuickGene-810 (Fujifilm, Singapore) according to the manufacturer’s instructions. RNA quality and quantity were determined with NanoDrop 1000 Spectrophotometer (Thermo Scientific, Waltham, MA, USA) and Agilent 2100 BioAnalyzer (Agilent Technologies, Santa Clara, CA, USA). DNAse treatment was performed on all samples using DNAse I (Roche, Basel, Switzerland) and subsequently cDNA synthesis from 2 µg of total RNA was carried out using Transcriptor Universal cDNA Master (Roche) following the manufacturer’s protocols. Before analysis cDNA was diluted 1:10 with water.

### 3.4. Real-Time Quantitative PCR

Real-time quantitative PCR was performed in a 384-well format on LightCycler 480 (Roche) using LightCycler 480 SYBR Green I Master (Roche). The PCR reaction consisted of 0.75 µL of template cDNA, 0.6 µL of 2.5 µM primer mix, 2.5 µL of SYBR Green I Master and 1.15 µL of RNAse-free water in a final volume of 5 µL. Three technical replicates were performed for each sample. The following thermal cycling conditions were used: 10 min at 95 °C followed by 45 cycles of 10 s at 95 °C, 20 s at 60 °C and 20 s at 72 °C. Primers (Supplementary Table S1) were designed and validated as described previously [57]. Several candidate reference genes were selected from the study of Kosir *et al.* [57] and with the use of qBase software [58], *Hmbs*, *Utp6* and *Ppib* were determined to be most stably expressed across all analyzed samples. Analysis and normalization of the data was done according to Vandesompele *et al.* [59]. 5 randomly chosen samples per group were analyzed.

### 3.5. Total Sterol Extraction and GC-MS Analysis

For the extraction of total (*i.e.*, free and esterified) sterols, 100–300 mg of liver tissue was put into 10 mL of Folch solution (chloroform/MeOH, 2:1, *v/v*) for 24 h. Appropriate volume of the extract (an equivalent of 2 mg of liver tissue) was used for cholesterol determination. Two µg of internal standard, hexadeuterium-labelled(d_6_) cholesterol (Medical Isotopes Inc., Pelham, NH, USA), was added and the mixture roughly split in half for quantification of free and total cholesterol. For total cholesterol measurements hydrolysis in 1 M NaOH in 90% ethanol for 2 h at 65 °C was performed. The sterols were then extracted with 2 × 3 mL of cyclohexane, solvent evaporated under a stream of argon and the residue derivatized using 100 µL of TMS (pyridine/hexamethyldisilazane/chloromethylsilane, 3/2/1, *v/v/v*, all Sigma-Aldrich, St. Louis, MO, USA) at 60 °C for 30 min. After evaporation of the solvent, samples were dissolved in 200 µL of hexane prior to injection on GC-MS machine. Part of the extract for quantification of free cholesterol was first evaporated and then immediately derivatized with TMS as described above. For determination of other sterols 150 ng of both tetradeuterium-labelled(d_4_) lathosterol and d_6_-sitosterol (both Medical Isotopes Inc.) were added to the Folch extract equivalent of 40 mg of liver tissue and subsequent sample handling followed the procedure for total cholesterol. In the last step samples were dissolved in 70 µL of hexane. Due to interference of cholesterol peak in chromatograms, samples of mice on HFC diet were additionally 10 times diluted with hexane prior to analysis by GC-MS. The GC-MS analysis was performed according to the method developed by Acimovic *et al.* [60]. Free and total cholesterol were quantified with the use of d_6_-cholesterol and its respective standard curve. Plant sterols campesterol and sitosterol were quantified according to d_6_-sitosterol and all other sterols to d_4_-lathosterol. The amounts of total sterols are given as ng/mg of wet liver tissue.

### 3.6. Total Bile Acids Extraction and GC-MS Analysis

For the extraction of total bile acids from gallbladder bile, 2 µL of bile was diluted with 0.5 mL of 0.9% NaCl and hydrolyzed with 9 M KOH in 50% ethanol overnight at 120 °C. Hydrolysis products were transferred to a separating funnel with 20 mL of 50% ethanol, 20 mL of 0.9% NaCl was added and washed two times with 25 mL of diethylether. In this way the neutral steroids were removed with diethylether under basic conditions. The remaining water phase was acidified with 5 mL of 6 M HCl, 50 mL of diethylether was added, shaken and left to separate. The water phase was discarded and diethylether phase washed two times with 25 mL of water. Diethylether was then evaporated and the residue with bile acids methylated with 40 µL (trimethylsilyl)diazomethane (Sigma-Aldrich) in 100 µL MeOH and 400 µL toluene for 5 min at room temperature. After evaporation of the solvents, derivatization with TMS (see above) was performed and the samples dissolved in 200 µL of hexane.

The samples were run on Agilent HP 6890N gas chromatograph coupled with Agilent HP 5973 MSD quadropole mass spectrometer (Agilent Technologies) using electron impact ionization mode. 1 µL of sample was injected in splitless mode onto gas chromatograph and separation was performed using HP-ultra1 25 m × 200 µm × 0.33 µm capillary column (Scantec Lab AB, Partille, Sweden) and helium as carrier gas at a flow rate of 1 mL min^−1^. Temperature program of the column was as follows: 180 °C for 1 min, then raised to 220 °C with a rate of 20 °C min^−1^ and further raised to 290 °C with a rate of 3.3 °C min^−1^, where it was held for another 15 min. Finally the temperature was raised to 300 °C in 1 min and held there for another 1 min giving a total run time of 41 min.

Peaks of cholic, deoxycholic, chenodeoxycholic, α-muricholic, β-muricholic, ω-muricholic and ursodeoxycholic acid were identified according to their respective standards that were run separately. Area under the curve of a single BA was quantified relatively against the total area of all BA of interest in a given sample. Cholic and α-muricholic acid were not clearly separated so they were integrated together.

### 3.7. Statistical Analysis

Statistical analyses were performed using R programming language (R version 2.14.2). First, data were tested for the homogeneity of variances using Levene’s test and median for calculating the test statistic. If the variances were homoscedastic, differences between the groups were tested with Fisher’s Least Significant Difference (LSD) test and if they were heteroscedastic, two-sample independent t-test with Welch’s correction was applied. All calculated *p* values were adjusted for multiple comparisons with the False Discovery Rate (FDR) procedure. Error bars in graphs represent standard errors of mean (SEM). P values less than 0.05 were considered statistically significant.

## 4. Conclusions

There is emerging evidence that sex represents an important factor in NAFLD and other cholesterol-related diseases [5,9,10]. As aberrant cholesterol synthesis is one of the driving factors of NAFLD [61], our study sheds light on physiological differences in hepatic cholesterol metabolism between the sexes by examining the effects of no or high dietary cholesterol in 129/Pas × C57BL/6J mice. Gene expression data suggests more active SREBP-2 dependent transcription in females in the absence of dietary cholesterol. In mice, higher hepatic cholesterol could potentially be more detrimental in females than in males in terms of progression and severity of liver inflammation in NAFLD [62]. It has also been shown that free cholesterol loading in the mitochondria can sensitize hepatocytes in transition from simple steatosis towards steatohepatitis [63]. Interestingly, females show higher amounts of cholesterol precursors than males when challenged with dietary cholesterol. This suggests that the metabolic flux through the post-lanosterol part of cholesterol synthesis differs between the sexes with males more efficiently balancing the cholesterol load than females. Higher expression of cholesterogenic genes in the females might not result in sufficient protein translation and/or protein activity that might result in accumulating cholesterol intermediates. To a certain extent these intermediates can modulate their own synthesis, yet they probably also serve other biological functions as shown for FF-MAS and T-MAS [64]. Some of these intermediates are toxic when in excess [31] and could potentially play a role in pathophysiology of NAFLD.

Due to the complexity of cholesterol synthesis and its many ways of regulation, further research in the field of sex differences would demand more comprehensive approaches by combining genetic, proteomic and metabolic studies together with mathematical modeling. This is important for further understanding of the sex bias in model organisms and in humans, in connection to the control of dietary cholesterol and the cholesterol-linked diseases.

## Supplementary Materials

Supplementary materials can be accessed at: http://www.mdpi.com/1420-3049/18/9/11067/s1.
