# Supplementary material for: Sex Differences in the Hepatic Cholesterol Sensing Mechanisms in Mice
Source: Molecules. 2013 Sep 10;18(9):11067–85. doi: 10.3390/molecules180911067 (PMC6270450; doi:10.3390/molecules180911067)

Supplementary Materials

Table S1. Primer information with gene/enzyme descriptions and determined efficiencies. Fw–forward, Rv–reverse.

Gene symbol	Gene/enzyme description	NCBI accession no.	Primer sequence	Efficiency
Cholesterol synthesis genes				
Hmgcs1	3-hydroxy-3-methylglutaryl-Coenzyme A synthase 1	NM_145942.4	Fw: GCCGTGAACTGGGTCGAA<br>Rv: GCATATATAGCAATGTCTCCTGCAA	1.88
Hmgcr	3-hydroxy-3-methylglutaryl-Coenzyme A reductase	NM_008255.2	Fw: CTTGTGGAATGCCTTGTGATTG<br>Rv: AGCCGAAGCAGCACATGAT	1.83
Fdft1	farnesyl diphosphate farnesyl transferase 1	NM_010191.2	Fw: CCAAACAGGACTGGGACAAG<br>Rv: GACGAGAAAGGCCAATTCC	1.90
Sqle	squalene epoxidase/ monooxygenase	NM_009270.3	Fw: TCAACCCCAGTCCAGTTCTC<br>Rv: GACTCCTTCAGGTGCTCAGG	1.77
Lss	lanosterol synthase	NM_146006.1	Fw: GGCACCAATGGATCACAGAT<br>Rv: AAACTCAGGTCTGTGGTGTGC	1.81
Cyp51	lanosterol 14α-demethylase	NM_020010.2	Fw: ACGCTGCCTGGCTATTGC<br>Rv: TTGATCTCTCGATGGGCTCTATC	1.75
Tm7sf2	transmembrane 7 superfamily member 2/sterol-Δ^{14}- reductase	NM_028454.2	Fw: GCCTCGGTTCCTTTGACTT<br>Rv: ATCAGCAGGGCCAGGTTA	1.82
Sc4mol	sterol-C4-methyl oxidase-like	NM_025436.2	Fw: CGGAATTGTGCTTTTGTGTG<br>Rv: GCGGGTTGAGAGGAATATCA	2.01
Nsdhl	NAD(P) dependent steroid dehydrogenase-like /sterol-4α-carboxylate 3-dehydrogenase	NM_010941.3	Fw: TGCAGCTCTAGGTGGAAAGG<br>Rv: GAACGTCCAGAAAGGGATTG	2.00
Hsd17b7	hydroxysteroid (17β) dehydrogenase 7	NM_010476.3	Fw: GCTCACTGTGACACCGTACAA<br>Rv: CCGGTTTTTGGTGGAAGAG	1.94
Sc5d	sterol-C5-desaturase/lathosterol oxidase	NM_172769	Fw: CCTTAATGAAACACCCACAGTTT<br>Rv: CTCCAGCAGGAACAGTGAGAC	1.84
Ebp	phenylalkylamine Ca2+ antagonist (emopamil) binding protein/sterol-$\Delta^{8,7}$-isomerase	NM_007898.2	Fw: TGTCCTACAGCTTGTGGTGTCT<br>Rv: AATAAACGGGGTGGCCTATC	1.89
Dhcr7	7-dehydrocholesterol reductase	NM_007856.2	Fw: GCTTCAGGCAGGCACTTAGA<br>Rv: GGATTTCGAAGCCATCAGG	1.85
Dhcr24	24-dehydrocholesterol reductase	NM_053272.2	Fw: GGTCATGACGGACGACGTA<br>Rv: AGGGCTTGTAGTAACTGCCAAT	1.91

Table S1. *Cont.*

Gene symbol	Gene/enzyme description	NCBI accession no.	Primer sequence	Efficiency
Cholesterol-related genes				
Srebf2	sterol regulatory element binding transcription factor 2	NM_033218.1	Fw: GCGTTCTGGAGACCATGGA<br>Rv:ACAAAGTTGCTCTGAAAACAAATCA	1.62
Ldlr	low density lipoprotein receptor	NM_010700.3	Fw: AGGCTGTGGGCTCCATAGG<br>Rv: TGCGGTCCAGGGTCATCT	1.79
Lxrα	nuclear receptor subfamily 1, group H, member 3 (Nr1h3)/liver X receptor	NM_013839.4	Fw: ACTTCAGTTACAACCGGGAAGA<br>Rv: GCTCTGGAGAACTCAAAGATGG	1.91
Cd36	CD36 antigen	NM_001159558.1	Fw: AAAACGACTGCAGGTCAACA<br>Rv: CATTTCTGCTTTTTCATCACCA	2.00
Scarb1	scavenger receptor class B, member 1	NM_016741.2	Fw: TCAGAAGCTGTTCTTGGTCTGAAC<br>Rv: GTTCATGGGGATCCCAGTGA	1.77
Abcg5	ATP-binding cassette sub-family G, member 5	NM_031884.1	Fw: CTTACCCACGGTTCCTTTCA<br>Rv: ACGCATAATCACTGCCTGCT	1.72
Abcg8	ATP-binding cassette sub-family G member 8	NM_026180.2	Fw: AAGACGGGCTGTACACTGCT<br>Rv: AGTAGATGGGCATCGCGTAG	2.00
Bile acid synthesis genes				
Cyp7a1	cholesterol 7-alpha-monooxygenase	NM_007824.2	Fw: CAGGGAGATGCTCTGTGTTCA<br>Rv: AGGCATACATCCCTTCCGTGA	1.88
Cyp7b1	25-hydroxycholesterol 7-alpha-hydroxylase	NM_007825.4	Fw: CCGATTCTGCCGTCTCCTT<br>Rv: GCAGCCTTACTCTGCAAAGCTT	1.82
Cyp8b1	7-alpha-hydroxycholest-4-en-3-one	NM_010012.3	Fw: AAGGCTGGCTTCCTGAGCTT<br>Rv: AACAGCTCATCGGCCTCATC	1.71
Cyp27a1	sterol 26-hydroxylase	NM_024264.4	Fw: CCTCACCTATGGGATCTTCATC<br>Rv: TTTAAGGCATCCGTGTAGAGC	1.90
Reference genes				
Utp6	small subunit (SSU) processome component	NM_144826.3	Fw: TTTCGGTTGAGTTTTTCAGGA<br>Rv: CCCTCAGGTTTACCATCTTGC	1.82
Hmbs	hydroxymethylbilane synthase	NM_013551.2	Fw: TCCCTGAAGGATGTGCCTA<br>Rv: AAGGGTTTTCCCGTTTGC	1.73
Ppib	peptidylprolyl isomerase B	NM_011149.2	Fw: GGAGATGGCACAGGAGGAAA<br>Rv: CCGTAGTGCTTCAGTTTGAAGTTCT	1.84

Table S2. Fold change (FC) differences of expression of cholesterogenic genes. Significant p values are bolded. HFC – high-fat diet with cholesterol, HFnC–high-fat diet without cholesterol

**Gene symbol**	**HFC/HFnC**						**Females/Males**			
	**Females and males**		**Females**		**Males**		**HFnC**		**HFC**	
	FC	p value	FC	p value	FC	p value	FC	p value	FC	p value
Hmgcs1	0.105	**0.015**	0.098	**0.003**	0.120	0.164	2.257	0.053	1.837	0.87
Hmgcr	0.160	**0.002**	0.160	**0.003**	0.159	0.075	1.806	0.087	1.822	0.789
Fdft1	0.344	**0.011**	0.403	**0.018**	0.241	0.075	1.729	0.086	2.89	0.281
Sqle	0.072	**0.001**	0.076	**0.036**	0.067	**0.025**	1.158	0.705	1.315	0.657
Lss	0.245	**0.002**	0.244	**0.002**	0.247	**0.018**	1.432	0.158	1.413	0.744
Cyp51	0.176	**0.001**	0.159	**0.033**	0.197	**0.013**	1.296	0.45	1.047	0.882
Tm7sf2	0.318	**0.001**	0.39	**0.026**	0.241	**0.012**	1.077	0.796	1.745	0.534
Sc4mol	0.062	**0.014**	0.06	0.075	0.065	0.055	2.295	0.216	2.139	0.500
Nsdhl	0.100	**0.005**	0.104	0.067	0.095	**0.014**	1.819	0.276	2.004	0.616
Hsd17b7	0.223	**0.001**	0.219	0.055	0.229	**0.003**	1.505	0.306	1.439	0.442
Ebp	0.308	**0.012**	0.266	**0.001**	0.406	0.176	2.356	**0.004**	1.541	0.620
Sc5d	0.272	0.113	0.233	0.117	0.495	0.072	5.646	0.106	2.66	0.467
Dhcr7	0.253	**0.004**	0.235	**0.016**	0.272	**0.024**	1.027	0.93	0.887	0.919
Dhcr24	0.467	**0.001**	0.451	**0.001**	0.488	**0.008**	1.370	**0.045**	1.265	0.476

Figure S1. Gallbladder bile acid composition. Black columns represent male and white columns female mice. Error bars represent SEMs. $^{\cdot}$ $p < 0.1$; * $p < 0.05$; ** $p < 0.01$; *** $p < 0.001$.

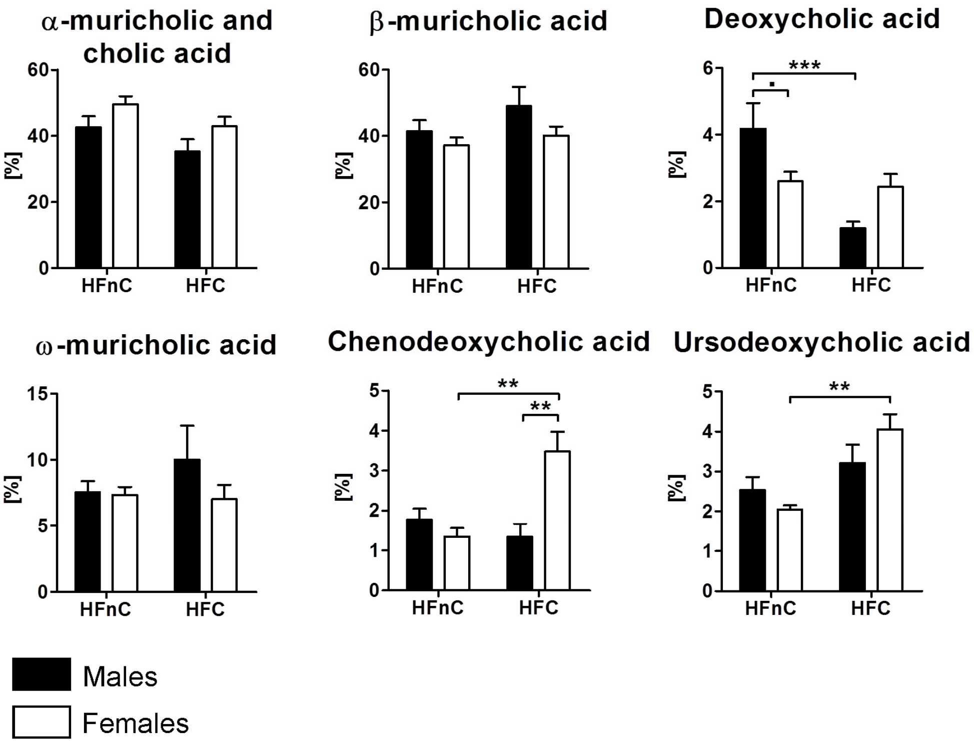

Supplement: Supplementary file 1 [file molecules-18-11067-s001.pdf]
